# Multiplexed profiling of GPCR activities by combining split TEV assays and EXT-based barcoded readouts

**DOI:** 10.1038/s41598-018-26401-9

**Published:** 2018-05-25

**Authors:** Sabrina Galinski, Sven P. Wichert, Moritz J. Rossner, Michael C. Wehr

**Affiliations:** 1 Molecular Neurobiology, Department of Psychiatry, Ludwig Maximilian University of Munich, Germany, Nussbaumstr. 7, Munich, 80336 Germany; 2 Systasy Bioscience GmbH, Adams-Lehmann-Str. 56, Munich, 80797 Germany

## Abstract

G protein-coupled receptors (GPCRs) are the largest class of cell surface receptors and are implicated in the physiological regulation of many biological processes. The high diversity of GPCRs and their physiological functions make them primary targets for therapeutic drugs. For the generation of novel compounds, however, selectivity towards a given target is a critical issue in drug development as structural similarities between members of GPCR subfamilies exist. Therefore, the activities of multiple GPCRs that are both closely and distantly related to assess compound selectivity need to be tested simultaneously. Here, we present a cell-based multiplexed GPCR activity assay, termed GPCRprofiler, which uses a β-arrestin recruitment strategy and combines split TEV protein-protein interaction and EXT-based barcode technologies. This approach enables simultaneous measurements of receptor activities of multiple GPCR-ligand combinations by applying massively parallelized reporter assays. In proof-of-principle experiments covering 19 different GPCRs, both the specificity of endogenous agonists and the polypharmacological effects of two known antipsychotics on GPCR activities were demonstrated. Technically, normalization of barcode reporters across individual assays allows quantitative pharmacological assays in a parallelized manner. In summary, the GPCRprofiler technique constitutes a flexible and scalable approach, which enables simultaneous profiling of compound actions on multiple receptor activities in living cells.

## Introduction

G protein-coupled receptors (GPCRs) are the largest and most investigated class of cell surface receptors transmitting the signal of numerous extracellular stimuli into the cell. Once a ligand binds to and activates a GPCR, heterotrimeric G proteins are activated. In turn, the G protein subunits dissociate to interact with effector proteins to initiate downstream signaling^1–3^. Prolonged receptor activation is determined by phosphorylation by one of several G protein-coupled receptor kinases (GRKs). The phosphorylation in turn promotes the binding of proteins of the β-arrestin family. The binding of β-arrestin sterically obstructs G protein coupling and triggers the desensitization and internalization of the GPCR^4,5^. Besides their function of GPCR desensitization, β-arrestins are also capable to act as adaptor initiating distinct signal transduction pathways^6–8^. Abnormal GPCR activities and consecutively deregulated signalling pathways also impact on the pathophysiology of various diseases, such as metabolic disorders, immune diseases, neurodegenerative diseases, and psychiatric disorders like schizophrenia and bipolar disorder^9,10^. As GPCRs have critical roles in the pathophysiology of many diseases, GPCRs are key drug targets. Hence, GPCRs are currently targeted by 33% of all marketed drugs, making them the largest druggable class of receptors^11^. Within the drug development process it is key to test the selectivity of a compound towards its destined target GPCR among various subfamilies^12^. To do this, the activities of multiple relevant GPCRs, i.e. the target GPCR as well as closely and distantly related GPCRs, must be monitored upon compound treatment.

For the analysis of GPCR activities and downstream signalling effects, a large number of tools were developed over the last decades utilizing several steps of the GPCR signalling cascade. Various biochemical and biophysical approaches based on the recruitment of G-proteins have been developed^13–16^. In addition, activities of GPCRs can also be monitored by the regulated recruitment of β-arrestin using genetically encoded biosensors. For example, GFP-tagged β-arrestin translocation upon receptor stimulation can be tracked by fluorescence imaging^17^. Recruitment of β-arrestin may also be measured using protein-protein interaction assays that utilize bioluminescence resonance energy transfer (BRET)^18^, fluorescence resonance energy transfer (FRET)^19^ or complementation strategies based on β-galactosidase^20^ or luciferase fragments^21^. Likewise, we have previously developed a similar β-arrestin2 recruitment GPCR activation assay based on TEV (tobacco etch virus) protease complementation. Notably, this proximity assay uses the artificial GAL4/UAS system and transcription-coupled reporters as readout, such as firefly luciferase^22,23^, and thus allows integration of molecular barcodes for multiplexed assays^24^. In this vein, we have previously amended the split TEV system to monitor receptor tyrosine kinase receptor activation in a truly multiplexed fashion by replacing the luciferase reporter gene with RNA-based molecular barcode reporters termed expressed tags (EXTs), which were analysed with custom-made microarrays^25,26^. GPCR activities may also be monitored using a β-arrestin2 induced proximity assay that is based on a full length TEV protease fused to β-arrestin2, which has been termed Tango^27^. Upon GPCR activation, the TEV protease cleaves off a transcription factor linked to the same GPCR to activate a reporter gene using the GAL4/UAS system^27^. This approach has been employed to develop a platform for parallelization of individual GPCR activation assays covering a large collection of the human GPCRome termed PRESTO-Tango^28^. Although PRESTO-Tango has been successfully applied to address the human druggable GPCRome, it still relies on individual assays performed in micro-well plates^28^. Here, we present a profiling tool termed GPCRprofiler that uses β-arrestin2 recruitment and combines split TEV-based GPCR activation assays with EXT molecular barcode reporters, which are quantified by next-generation sequencing. By introducing internal and external calibrators, we can simultaneously perform dose-response analyses of 19 GPCRs using pools of single readouts. Thus, our approach substantially reduces time and costs and has the potential to be scaled up to the GPCRome level.

## Results

### Combining GPCR split TEV assays and EXT-based barcode readout for multiplexed assays

**Figure 1:**
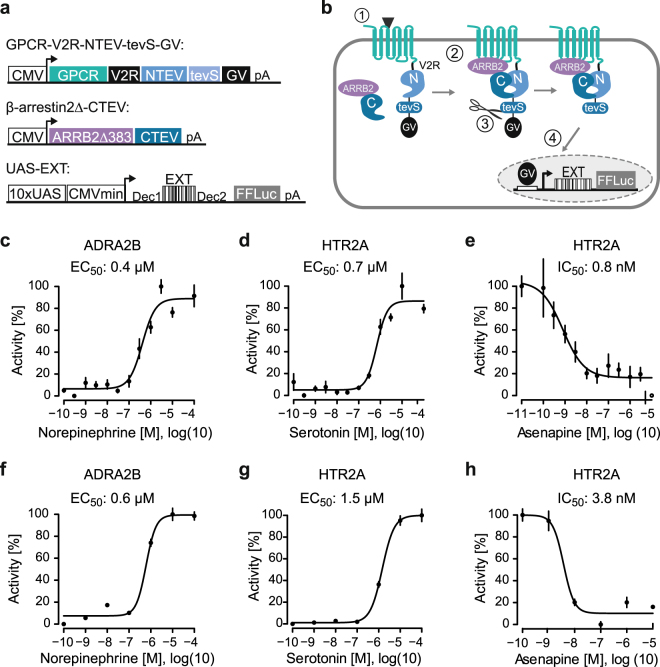
Constructs and assay principle to profile GPCR activities using luciferase and barcode reporters. (**a**) Modular design of split TEV and reporter constructs. GPCR-V2R-NTEV-tevS-GV constructs are composed of the GPCR fused to the C-terminal domain of the arginine vasopressin receptor 2 (V2R) followed by the N-terminal TEV protease fragment (NTEV, aa 1–118), the TEV cleavage site (tevS) and the GAL4-VP16 transcription factor (GV). β-arrestin2delta-CTEV constructs harbours the truncated human β-arrestin2 (ARRB2delta383, aa 1–383) fused to the C-terminal TEV protease fragment (CTEV, aa 119–221). Both fusion proteins are expressed under the control of a cytomegalovirus promoter (CMV). UAS-EXT reporter constructs are composed of 10-fold clustered upstream activating sequence elements (UAS), a CMV minimal promoter followed by unique EXT barcode sequences and the firefly luciferase reporter gene. EXT sequences are flanked by primer binding sequences (Dec1 and Dec2). See Supplementary Table S1 for details on human GPCRs and EXT assignment. See Supplementary Table S2 for EXT barcodes. (**b**) Schematic representation of GPCR split TEV assays. The ligand-dependent receptor activation results in the recruitment of β-arrestin to the GPCR (1) and the reconstitution of the protease activity (2). TevS is cleaved (indicated by the scissors) (3) and the released GV translocates to the nucleus to activate the transcription of EXT barcode reporters and the firefly luciferase gene by binding to UAS elements (4). (**c–h**) Dose-response curves of the ADRA2B adrenergic receptor and HTR2A serotonin receptor in luciferase-based (**c–e**) and EXT-based (**f–h**) split TEV assay. ADRA2B-V2R-NTEV-tevS-GV and HTR2A-V2R-NTEV-tevS-GV plasmids were transfected into β-arrestin2delta-CTEV stable expressing PC12 cells and stimulated with increasing concentrations of norepinephrine, serotonin or asenapine. The asenapine stimulation was followed one hour later by 0.7 µM serotonin. Data are shown as mean ± s.e.m. (error bars), n = 6.

GPCR split TEV assays allow monitoring ligand-dependent GPCR activities using the stimulation-dependent interaction of β-arrestin2 and a candidate GPCR. The split TEV technique is based on the complementation of two inactive fragments of the TEV protease (NTEV and CTEV) to reconstitute the functional protease due to an occurred protein-protein interaction^22^, in this case a GPCR and β-arrestin2. To do this, the NTEV fragment and the artificial transcription factor GAL4-VP16 (GV) were fused to the C-terminal end of a candidate GPCR, separated by a TEV cleavage site (tevS) (Fig. 1a,b). To improve the stability of the interaction between a given GPCR and the arrestin, a short sequence from the C-terminal end of the human arginine vasopressin receptor 2 (AVPR2) (abbreviated V2R) was introduced between a candidate GPCR and NTEV^17,27^. V2R contains multiple phosphorylation sites for GRKs. These sites provide, when phosphorylated, enhanced binding of β-arrestin. The CTEV fragment was fused to human truncated β-arrestin2 lacking the entire C-terminal tail (ARBB2 lacking amino acids 383–410) (Fig. 1a). This truncation exhibits stronger stimulation-dependent receptor desensitization and therefore enhances the assay sensitivity compared with wild type β-arrestin^23,29^. The CTEV fragment carries the stabilizing point mutation S219P^30^ and is truncated after amino acid 221 to remove the auto-inhibitory C-terminal tail^31,32^.

After ligand-dependent GPCR activation, β-arrestin is recruited to the receptor leading to the reconstitution of the TEV protease causing the release and translocation of GV into the nucleus, where GV binds and activates a transcriptional reporter (Fig. 1b). To simultaneously monitor receptor activation of multiple GPCRs within one experiment, we generated 85 EXT reporters that are amenable to multiplexed assays (Supplementary Tables S1 and S2). EXTs are RNA reporters harbouring unique barcode sequences, which replace standard reporter proteins and can be analysed in a pooled experimental setting^25^. EXT reporters are placed upstream of the firefly luciferase gene and their expression is under the control of a CMV minimal promoter and clustered GAL4-responsive upstream activating sequence (UAS) elements (Fig. 1a,b). Because the reporters are designed to drive the expression of EXT barcodes and firefly luciferase, they can be used in multiplexed EXT assays or in single luciferase assays.

In a first test, we investigated the functionality of single GPCR split TEV assays coupled to an EXT readout and compared its performance to standard luciferase readings. By monitoring only one GPCR per assay, we compared adrenergic receptor ADRA2B and serotonin receptor HTR2A activity in independent single luciferase and single EXT assays using dose-response analyses (Fig. 1c–h). For these assays, cells were transiently transfected with GPCR split TEV components and reporters, which were allowed to express for 20 h. Following to a starvation period of 18 h, cells were stimulated with agonists for 6 h and lysed for subsequent luciferase assays or EXT-based next-generation sequencing analysis (Supplementary Fig. S1a). For antagonistic assays, compounds were added 1 h before agonist to ensure proper incubation (Supplementary Fig. S1b). For clarity, varying experimental conditions, such as plate formats, for applied assay formats are summarized in Supplementary Table S3. Epinephrine stimulation resulted in similarly comparable dose-dependent activation of ADRA2B with EC_50_ values of 0.4 µM in luciferase and 0.6 µM in EXT-based assay (Fig. 1c,f). Stimulation of HTR2A with serotonin resulted in a dose-dependent activation in luciferase and EXT-based assays, with EC_50_ values of 0.7 µM and 1.5 µM, respectively (Fig. 1d,g). Likewise, an inhibition using the antagonist asenapine in the presence of serotonin caused a dose-dependent decrease in activity, with an IC_50_ of 0.8 nM in luciferase and 3.8 nM with EXT-based readouts (Fig. 1e,h). We thus conclude that EXT-based readouts deliver highly similar response profiles compared to the luciferase standard.

### Design of multiplexed GPCR profiling assays

**Figure 2:**
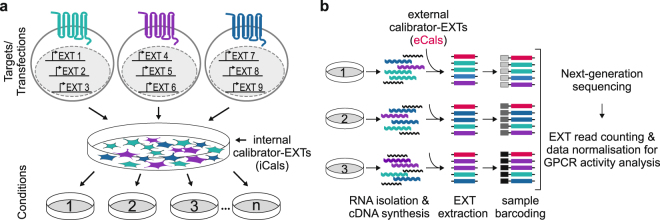
Design and strategy of multiplexed EXT-based split TEV GPCR activation assays. (**a**) Design of multiplexed GPCR profiling assay. For the mix & divide design, a separate transfection of GPCR-V2R-NTEV-tevS-GV, β-arrestin2delta-CTEV and three unique EXT reporter plasmids was done for each GPCR. After 2 h, all transfections were pooled with an internal calibrator barcode mix (iCals), divided into assay samples and plated onto 6-well plates. Samples were treated under different conditions depending on the assay. (**b**) Processing of samples after cell lysis. From each sample, the total RNA was isolated and converted into cDNA. After adding external calibrator barcodes (eCals), EXT sequences were PCR-amplified and each sample was additionally barcoded (Supplementary Fig. S3). Afterwards, samples were pooled and sequenced using next-generation sequencing followed by sample splitting, read counting, sample normalization, and calibration.

The general experimental setup of multiplexed GPCR profiling assays is composed of separate in-solution transfections of single GPCR split TEV components (GPCR-V2R-NTEV-tevS-GV and β-arrestin2delta-CTEV) and EXT reporters. Three unique EXT reporters were assigned to each GPCR (assay EXTs) (Supplementary Table S1). In parallel, two to six control EXT reporters were transfected separately at the same concentration without any GPCR, and served as internal calibrators (iCals). Two hours after transfection, all cell populations were pooled, mixed, and evenly divided into fractions for plating onto assay dishes (Fig. 2a). The cells were cultured and stimulated under identical conditions for agonist or antagonist assays as initially described for GPCR split TEV assays (Supplementary Fig. S1a,b). To determine the optimal assay conditions, we performed online-luciferase split TEV measurements for selected dopamine-, serotonin- and β-adrenergic receptors to follow the kinetics of the approach (Supplementary Fig. S1c). Based on these analyses, in all following experiments cells were harvested 6 h after compound addition (Supplementary Fig. S1d). As we have applied transient transfections, each GPCR has a different steady-state of newly synthesized vs. already present receptor proteins, causing an individual pattern of activation and desensitization. The best overlap among activations of GPCRs tested in the online-luciferase assays was obtained at 6 h post stimulation, which was set as general time point for lysis of GPCRprofiler experiments.

After cell lysis, RNA from each sample was isolated and converted into cDNA by reverse transcription. To control the next steps of amplification and sequencing, additional control EXT reporters were added as plasmids to each sample to represent low- and higher-level expressed EXT reporters within all data points of each individual assay and served as external calibrators (eCals) (Fig. 2b). For quantitative analysis all EXTs (assay EXT reporter, iCal EXTs, and eCal EXTs) were simultaneously PCR-amplified and each sample was labelled with an additional unique sample barcode (Supplementary Fig. S2). This permits the mixing of all samples and sequencing of all pooled samples using a single sequencing run (Fig. 2b). The sample barcodes enable tracing experimental conditions, whereas the read counts per EXTs serve as quantitative measurements for the corresponding GPCRs. To analyse the biological output of the EXT assays, the raw sequencing data were processed by code splitting, read counting, and sample normalization and calibration steps (for details see Methods). From the normalized data the fold changes of receptor activities were finally calculated.

### Multiplexed profiling of GPCR activation

We applied the GPCRprofiler assay to simultaneously assess ligand-dependent activation of multiple GPCRs. We selected a panel of 19 GPCRs of different aminergic and peptide-binding subfamilies. Prior to this selection, the functionality of our candidate GPCRs was individually tested in standard luciferase split TEV assays to assess background activity and stimulation efficiency (Supplementary Fig. S3). Notably, for all our candidate GPCRs except ADRB3 successful β-arrestin recruitment assays are reported^28,33^. For ADRB3, we show in this study that stimulation of this GPCR, if modified by the V2R tail, can be monitored using a split TEV-based beta recruitment assay (Supplementary Fig. S3). In these assays, GPCR activation was assayed both in U2OS and PC12 cells, and GPCRs that displayed a significant activation in at least one cell line when stimulated with their cognate ligand were chosen for the GPCRprofiler assays.

**Figure 3:**
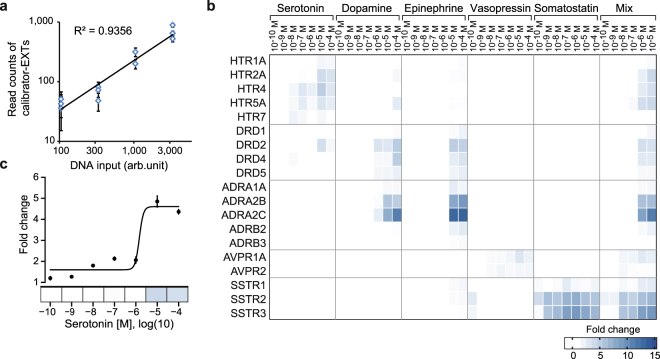
Multiplexed profiles of agonist-induced GPCR activities. (**a**) Sequencing reads per input plasmid molecules of different external calibrator EXTs reveal a linear distribution with high correlation (R^2^ > 0.93). (**b**) Heatmap of multiplexed EXT-based measurement of GPCR activations. Plotted are fold changes of receptor activation in reference to unstimulated condition of 19 different GPCRs in U2OS cells. All GPCRs were treated with different concentrations of the single compounds serotonin, dopamine, epinephrine, vasopressin and somatostatin and a mixture (mix) of the five compounds. (**c**) Individual EXT-based dose response analysis of the serotonin receptor HTR2A stimulated by serotonin extracted from multiplexed GPCR assay showing concentration dependent receptor activation.

For the GPCRprofiler, each GPCR was assigned to three unique EXT reporters (Supplementary Table S1 and S2). All receptors were treated with serotonin, dopamine, epinephrine, vasopressin, and somatostatin separately within a concentration range of 100 pM to 100 µM at single log-scale steps. In addition to the stimulation with the single compounds, a compound-mixture (mix) of all agonists was tested. The first set of experiments were carried out in the human osteosarcoma cell line U2OS. We normalized the raw sequencing data to total read numbers of sample codes, followed by averaging reads of internal and external calibrator EXTs as described above (Supplementary Fig. S4). The read counts of external calibrator EXTs showed a linear increase to the increasing level of input with high correlation (R^2^ > 0.93), indicating appropriate calibration and sensitivity measures for this multiplexed approach (Fig. 3a). The response profiles for each receptor at each stimulus condition were visualized in a heatmap, covering the five selected GPCR ligands, the ligand mix, and 19 GPCRs (Fig. 3b). For each GPCR, a dose-dependent receptor activation induced by its cognate agonist was observed, as exemplified by the HTR2A dose-response data extracted from the profiling experiment (Fig. 3c). In this vein, the five serotonin receptors (HTR1A, HTR2A, HTR4, HTR5, and HTR7) were activated by serotonin up to 2–5-fold (Fig. 3b). The dopamine and the adrenergic receptors displayed maximal induction rates ranging from 2-fold for DRD1, DRD3 and ADRB3, and up to 10- and 13-fold for ADRA2B and ADRA2C. Notably, both receptor subfamilies exhibited elevated signals upon dopamine as well as epinephrine stimulation. The dopamine receptor DRD2 was 5-fold activated at 10 µM of serotonin. Vasopressin receptors AVPR1A and AVPR2 were induced by vasopressin. The induction rates of the somatostatin receptors by somatostatin could be detected for SSTR1 up to 4-fold and for SSTR2 and SSTR3 up to 9–10-fold. When all GPCRs were stimulated with the ligand mix, analogous dose-dependent activation signals were produced for all GPCRs. Importantly, signals were highly similar in their intensities to the observed signals in the corresponding single agonist conditions (Fig. 3b).

To test whether the GPCR profiler identifies different response profiles in other cell types, we performed an identical experiment in PC12 cells. Activation profiles for adrenergic, vasopressin, and somatostatin receptors are comparable in both cell types (Supplementary Fig. S5a). In contrast, profiles for serotonin and dopamine receptors displayed varying efficiencies. For example, activities for HTR2A, HTR4, and DRD1 were readily monitored in PC12 cells, but not for the other serotonin (HTR1A, HTR5A, HTR7) and dopamine receptors (DRD2, DRD4, DRD5) tested. When comparing fold changes obtained from GPCR profiles in U2OS and PC12 cells, we noted that activities for selected GPCR, such as HTR2A, ADRA1A, and AVPR1A, are more prominent, as evident from 89.5-fold, 314.2-fold, and 44.3-fold activation, respectively (Supplementary Fig. S5b). However, U2OS cells are more applicable to the GPCR profiler, as a larger number of functional GPCR activity assays could be performed simultaneously. For HTR7 and ADRB3, however, no consistent activation dependent increases in assay activity were observed in U2OS and PC12 cells, neither by applying individual ligands nor by using the agonist mix suggesting that these two assays are not functional or below detection limit. Therefore, these two assays were excluded from any further antagonist experiments. In summary, the results suggest that the used agonists, which are classical endogenous neurotransmitters and hormones, are acting specifically on their cognate GPCR subgroups. For dopamine and epinephrine treatments, however, we observed substantial cross-talk between their cognate receptor subfamilies, which is in agreement with published data^34–37^.

### Profiling GPCR activities induced by antipsychotic drugs

**Figure 4:**
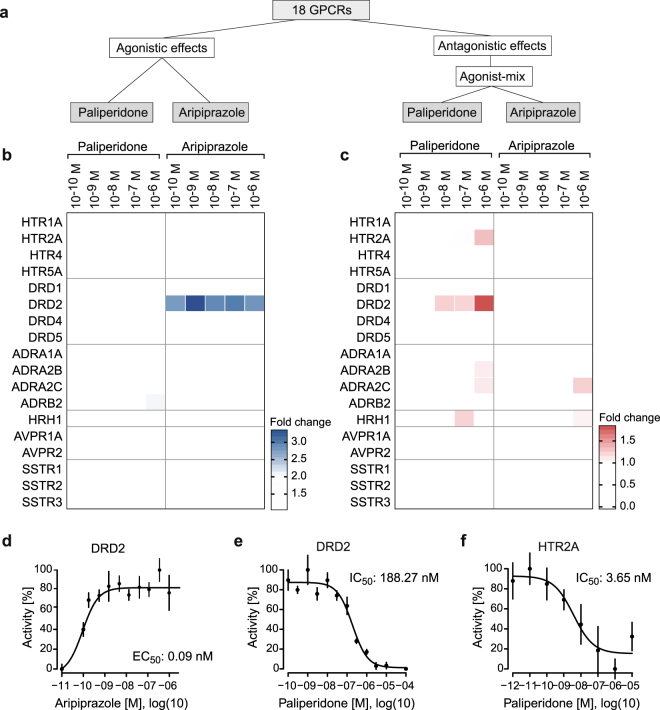
Multiplexed GPCR activity profiles induced by antipsychotic drugs. (**a**) Graphical representation of stimulation conditions in compound profiling GPCR assay monitoring effects of the antipsychotic drugs paliperidone and aripiprazole. GPCRs were stimulated with antipsychotic drugs alone to monitor receptor activating agonistic compound effects and in combination with agonist-mix to detect antagonistic receptor inhibiting compound effects. (**b**) Heatmap of agonistic compound effects. GPCRs were stimulated with paliperidone and aripiprazole at the indicated concentrations. (**c**) Heatmap of antagonistic compound effects. GPCRs were stimulated with drugs as indicated together with agonist-mix containing the agonists serotonin, dopamine, epinephrine, histamine, vasopressin and somatostatin each at 1 µM. Agonist-mix was added 1 h after drugs. Validation of drug induced effects by dose response curves of standard single luciferase GPCR split TEV assays of (**d**) receptor activation of the dopamine receptor DRD2 by aripiprazole, (**e**) receptor inhibition of the dopamine receptor DRD2 and (**f)** the serotonin receptor HTR2A by paliperidone.

Next, we applied the multiplexed GPCRprofiler to measure the effects of GPCR targeting drugs on receptor activity. Here, 18 different GPCRs were treated with the antipsychotic drugs paliperidone and aripiprazole (Fig. 4a). Paliperidone (9-OH-risperidone) is the active metabolite of the older atypical antipsychotic risperidone and acts in the classical mode of action of antipsychotics to inhibit DRD2 and HTR2A receptors. In addition, binding affinities to α-adrenergic receptors and the histamine receptor HRH1 were described for paliperidone using biochemical assays^38–40^. In contrast, aripiprazole is controversially described either as DRD2 partial agonist or, depending on the cell type used, as functionally selective drug acting as DRD2 agonist, partial agonist, or antagonist^41,42^. To address any changes in GPCR activities caused by the selected drugs, receptors were stimulated for 6 h applying two different parameters. First, to monitor agonistic effects, GPCRs were treated with the drugs only (Supplementary Fig. S1a). Second, to measure antagonistic effects, GPCRs were treated both with the drugs and the corresponding agonist (Supplementary Fig. S1b). This experimental design therefore allows to identify potential antagonistic effects of agonists and vice versa. For a profiling experiment, drugs were applied at increasing concentrations. After a 1 h pre-incubation period, an agonist-mix of serotonin, dopamine, epinephrine, histamine, vasopressin, and somatostatin was added, with a concentration of 1 µM each (Supplementary Fig. S1b). Thus, for the two stimulation parameters, the obtained signals either result from direct drug-induced receptor activation, suggesting the drug acting as agonist. Alternatively, a drug-induced inhibition of agonist-mediated GPCR activation specifies the drug as antagonist.

Both strong and subtle modulatory effects for agonistic and antagonistic actions were scored. For strong agonistic effects, we visualized conditions that displayed a minimum fold change of 2. To also detect subtle agonistic effects (i.e. less than a 2-fold change of activity), we calculated percent-wise changes for each step of increasing drug concentrations. We determined a positive effect to be true if a minimum of three consecutive increases were scored. Strong agonistic effects were observed for aripiprazole on DRD2, showing a constantly high activation for all concentrations applied (Fig. 4c). Using standard single GPCR luciferase assays, we confirmed the agonistic effect caused by aripiprazole (Fig. 4d). Further, subtle agonistic effects for aripiprazole were detected on the serotonin receptor HTR2A in a dose-dependent manner (Supplementary Fig. S6).

For strong antagonistic effects, fold changes for each condition were subtracted from the corresponding maximal receptor activity within one dose-response treatment, and inverted fold change values above 1 were visualized. Both drugs showed dose-dependent receptor inhibition on various GPCRs. Paliperidone showed dose-dependent inhibition of receptor activity on several GPCRs, i.e. the dopamine receptor DRD2 (Fig. 4c,e), the serotonin receptor HTR2A (Fig. 4c,f), the two α-adrenergic receptors ADRA2B and ADRA2C and the histamine receptor HRH1 (Fig. 4c). Likewise, aripiprazole inhibited the α-adrenergic receptor ADRA2C and histamine receptor HRH1.

## Discussion

We describe a sensitive, robust and multiplexed assay, termed GPCRprofiler, to monitor multiple GPCR activities in a parallelized manner by combining a β-arrestin2 recruitment assay with a transcriptional barcode reporter strategy. Single GPCR activity assays are based on the protein-protein interaction technique split TEV that allows to robustly measure the regulated association of activated (i.e. phosphorylated) receptors with β-arrestin2. As the split TEV method allows the flexible use of transcriptional reporters, we complemented the standard firefly luciferase reporter used for single assays with RNA barcode reporters, which allow an integration into multiplexed cell-based assays. Using this multiplexed approach, we simultaneously addressed the activation of 19 selected GPCRs from five sub-families (serotonin, dopamine, adrenergic, vasopressin, and somatostatin receptors) with their cognate ligands (i.e. serotonin, dopamine, epinephrine, vasopressin, somatostatin, and in a mix containing all ligands) in a cross-wise manner. Notably, the activity profiles for dopamine and epinephrine were largely overlapping for dopamine and adrenergic receptors. In addition, dopamine treatment lead to an activation of the serotonin receptor HTR2A in both U2OS and PC12 cells, suggesting that dopamine may have some promiscuous properties^43^. However, serotonin receptor HTR5A was activated by dopamine in U2OS cells only, possibly due to cell type specific effects^44^.

For the analysis of drugs, it is critical to assess its functional properties, as a drug may act as agonist, partial agonist, or antagonist. Therefore, monitoring these diverse drug effects in GPCR profiling assays requires a flexible setup that allows measuring agonistic and antagonistic effects in separate experimental paradigms that can be, however, performed and analysed in parallel. In our profiling approach, we applied a co-treatment paradigm using an agonist mix of ligands that keeps numbers of samples low when compared to applying the agonists separately. We challenged the GPCR profiler with the two antipsychotic drugs paliperidone and aripiprazole to assess the performance of our approach.

Paliperidone, the primary active metabolite of risperidone, is a classical atypical antipsychotic drug that acts predominantly as antagonist. The monitored inhibitory effects on HTR2A, DRD2, the α-adrenergic receptors and HRH1 are consistent with the literature^39,45^. Aripiprazole is reported to act functionally selective as agonist and antagonist depending on both the receptor and cell type^42^. Aripiprazole exerted agonistic effects on DRD2 and HTR2A, but antagonistic effects on HRH1, and these findings were validated in single luciferase assays. The activation of HTR2A is contrarily discussed in the literature. Commonly, an antagonism for this receptor is described^46^. However, a weak partial agonistic effect was reported for aripiprazole in GF62 cells^42^. It may be possible that aripiprazole acts functionally selective not only on DRD2^41^ but also on HTR2A in a cell type-dependent manner. For aripiprazole, an additionally partial agonism on the serotonin receptor HTR1A is reported, which was, however, not detected in this multiplexed GPCR assay, possibly caused by a limited sensitivity of the assay in U2OS cells. A comparison of our findings using the GPCRprofiler versus public databases and literature shows consistent overlap (Supplementary Table S4).

Several methods are available to assess ligand actions on GPCRs. The most conventional approach to identify ligand receptor interactions are cell-free binding assays. These assays have the intrinsic disadvantage that compounds are frequently radioactively labelled, and that binding affinity data does not provide any information on the functional properties of ligands. Thus, it is neither clear whether ligands act as agonist or antagonist, nor which efficacies are exerted under physiological conditions^16^. These limitations are overcome in cell-based functional assays. These approaches address ligand-dependent receptor activation and signalling within a cellular context. Most of these approaches rely on receptor-mediated activation of G proteins, either by directly measuring of second messenger levels (e.g. cAMP and calcium)^47–49^ or by using reporter gene assays that respond to second messenger activities^50^. Monitoring multiple GPCR-ligand interactions within one experimental setup was, however, not feasible as it is known that different G proteins couple to various types of GPCRs. A sensitive and robust method to monitor receptor activation of GPCRs uses the dynamic recruitment of β-arrestin, which is a measurement independent of G protein activity^27,28^. In addition, a recent study supports this view as β-arrestin can be recruited to activated GPCRs in the absence of active G proteins^51^. Recently, a large collection of cell-based assays using β-arrestin recruitment was introduced to interrogate the druggable GPCRome in a parallel manner^28^. The assay uses a modified Tango assay^27^ for screening compound libraries against a multiplicity of orphan and non-orphan GPCRs. However, the approach is based on single compound per target assays. Therefore, when for example analysing target selectivity for a given drug on multiple GPCRs, screening of GPCR activities will lead to extensive consumption of materials at increasing costs.

The GPCR profiling assay we introduce provides the following benefits: (I) the GPCRprofiler enables the rapid and easy detection of multiple GPCR activities in response to several ligands within one experiment. The GPCRprofiler monitors GPCR activation at a time point that is defined by optimal performance of individual GPCR assays. As the readout is based on molecular barcodes, large amount of data can be obtained for one compound within one measurement. This stands in contrast to conventional single assays, wherein each receptor-ligand combination must be measured separately. Therefore, our approach may be of advantage when compounds are limited e.g. in early phases of drug discovery programs. (II) The presented multiplexed GPCR assay is highly flexible and scalable in terms of combinations of receptors and ligands using the mix & divide design. The GPCRprofiler provides sensitive and specific activity readouts using transient transfections, enabling the addition of new GPCR activity assays easily. However, stable cell lines expressing the receptor and/or adapter protein might enhance the robustness and sensitivity of the assay, especially for weak receptor activations caused by partial agonists that may require a close-to-endogenous expression of GPCRs. (III) The GPCRprofiler can be principally performed in all cell lines and primary cells that are amenable to transient transfections allowing to screen for cell type-dependent effects of compounds.

GPCR recruitment assays based on β-arrestin2, like split TEV-based GPCR activity assays, use tagged GPCRs, and it may be possible that modified receptors show altered signalling properties^52^. For split TEV, it has been shown that GPCR-NTEV-tevS-GV fusions do not substantially interfere with assay performance and signalling^23^. Split TEV-based GPCR recruitment assays are designed to monitor receptor activation only, but not downstream signalling effects.

However, the GPCRprofiler may be complemented with genetically encoded pathway reporter assays that use overexpressed native GPCRs and molecular barcodes as final readout^25^. Using this approach, GPCR target profiling can be combined with cellular pathway profiling to provide a holistic view of compound actions complementary to biochemical or transcriptomic drug profiling^53^.

In summary, we present a multiplexed GPCR activity assay that uses a β-arrestin recruitment assay and molecular barcoding as readout to assess multiple GPCR activities in parallel. We validated the GPCRprofiler using cognate ligands and challenged the assay using the antipsychotic drugs paliperidone and aripiprazole. As the GPCRprofiler can be flexibly expanded by additional targets, it therefore might represent a suitable platform technology for early drug discovery.

## Methods

### Plasmids

All expression plasmids were generated by Gateway recombination cloning (Invitrogen). ORFs of GPCRs were PCR amplified from a mix of human heart, liver and brain cDNA library using proofreading polymerases (PfuUltra, Stratagene or Pwo, Roche). DRD4 ORF was purchased from Bio Basic Inc. as synthesized linear sequence. ADRB3 ORF shuttle clone was purchased from Source Bioscience (IOH29805). The entry vector for β-arrestin2delta383 was described before^23^. All ORFs were BP-recombined into pDONR plasmid and the yielded Entry vectors were sequence verified. Entry vectors were then LR-recombined into the split TEV destination vectors pTag4C_X-V2R-NTEV-tevS-GV or pcDNA3.1_X-CTEV, plasmids were described in detail before^22,23^, to obtain the final expression vectors of GPCR-V2R-NTEV-tevS-GV and β-arrestin2delta-CTEV fusion constructs. EXT reporter gene plasmids were generated by three-fragment multisite Gateway recombination (Invitrogen). EXT synthesis and entry vectors were described before^25^. To yield final reporter constructs Entry vectors encoding ten-time clustered GAL4-responsive cis-elements (UAS, attL1/attL4), a CMV minimal promoter (attL4/attL3) were LR-recombined with an EXT entry vector library (attL3/attL2) to obtain final 10xUAS-CMVmin-EXT reporter library. All constructs were sequence verified.

### Compounds

Serotonin hydrochloride, Histamine dihydrochloride, Asenapine maleate were purchased from Tocris, Dopamine hydrochloride, Epinephrine hydrochloride, [Arg8]-Vasopressin acetate salt, Paliperidone, Somatostatin were obtained from Sigma-Aldrich, Aripiprazole was purchased from Toronto Research Chemical.

### Cell culture

U2OS cells were maintained in McCoy’s 5A medium (Gibco) including GlutaMAX (Gibco), supplemented with 10% fetal bovine serum (FBS, Gibco) and 100 U/ml each of penicillin and streptomycin (Lonza). PC12-tetOFF cells were maintained in low glucose DMEM medium (1 g/L, Lonza), supplemented with 10% FBS, 5% horse serum (HS, Gibco), 2 mM GlutaMAX (Gibco) and 100 U/ml each of penicillin and streptomycin. PC12 cells were grown on poly-L-lysine (Sigma) coated surfaces for maintenance and experiments; U2OS cells were grown on poly-L-lysine surfaces only for experiments. All cells were grown at 37 °C and an atmosphere of 5% CO_2_.

### Luciferase reporter assays

For luciferase assays cells were plated on 96-well white plates at 4 × 10^4^ cells/well (PC12) one day before the experiment. The assays were performed with 6 wells as replicates per condition. Cells were transfected with split TEV and EXT reporter plasmids using lipofectamine 2000 (Invitrogen). Plasmids and lipofectamine 2000 transfection reagent (Invitrogen) were diluted in opti-MEM (Gibco) and incubated for 20 min at room temperature. Medium was removed from cells, the transfection-mix was added and the plates incubated for 2 h at 37 °C, followed by the adding of double-volume of culture medium to dilute transfection reagents. On the next day, culture medium was exchanged by serum-reduced assay medium (U2OS: McCoy’s 5 A supplemented with 0.5% dialyzed FBS (Gibco), 0.1 mM NEAA (Gibco); PC12: low glucose DMEM (1 g/L) supplemented with 1% dialyzed FBS and 0.1 mM NEAA) to induce starvation of the cells for 17–18 h. On day 3, medium was removed and cells were treated with compounds diluted in assay medium at different concentrations for 6 h at 37 °C. After 6 h of compound treatment the medium was removed and the cells were lysed with 30 µl passive lysis buffer (Promega). The reading of firefly luciferase was carried out with a Mithras LB 940 Microplate Reader (Berthold Technologies) and the software MicroWin2000. The data was exported from MicroWin2000 to Excel and the mean and standard error of the mean (s.e.m.) of the 6 replicates for the firefly readings were calculated.

### Transfection and stimulation conditions in single EXT reporter assays

For single EXT reporter assays cells were plated on 6-well plates at 4 × 10^5^ cells/well (PC12) one day before the experiment. The assays were performed in duplicates for each stimulation condition. Cells were transfected as described for luciferase assays. After 2 h of incubation, the transfection mix was removed from the cells and culture medium added and incubated for 20 h at 37 °C. Cells were then starved by medium change to serum-reduced assay medium. Afterwards, compounds at different concentrations were applied in duplicates for 6 h at 37 °C. After compound treatment cells were washed with PBS and lysed with 500 µl RLT buffer (Qiagen). Lysates were processed for sequencing as described below.

### Transfection and stimulation conditions in multiplexed EXT reporter assays

For EXT assays transfections were performed in-solution using lipofectamine 2000. Cells were resuspended in culture medium without antibiotics at a density of 1.5 × 10^6^ cells/ml (U2OS) or 3 × 10^6^ cells/ml (PC12). Split TEV and EXT-reporter plasmids and lipofectamine 2000 were mixed and incubated in serum-reduced opti-MEM for 20 min at room temperature. Transfection-mixes were added to cell suspensions and incubated for 2 h at 37 °C without shaking. Suspensions were centrifuged for 5 min at 900 rpm and resuspended in culture medium to remove transfection reagents. All transfection samples were combined within one pool and plated on multiple wells of 6-well plates and incubated for 24 h at 37 °C. Afterwards, cells were starved for 17–18 h and then treated with compounds for 6 h in duplicates. After compound treatment cells were washed with PBS and 500 µl RLT lysis buffer was applied for cell lysis. Lysates were processed for sequencing as described below.

### Sequencing library preparation and next-generation-sequencing of EXT assays

For sequencing single and multiplexed EXT assays RNA was purified from cell lysates over RNeasy columns (Qiagen) including on-column DNase treatment according to the manufacturers’ protocol (RNeasy Mini Kit, Qiagen) and eluted in 50 µl H_2_O. RNA was additional in solution treated with Turbo DNase (Ambion) for 30 min at 37 °C and repurified over an RNeasy column. First-strand cDNA synthesis was performed with 1 µg total RNA using superscript III reverse transcriptase (Invitrogen) and 120 pmol of a random nanomer primer. For later data normalization, additional EXT reporter plasmids were added as a mix of twelve external calibrator EXTs (eCals) to each sample. Single eCals were added at semi-logarithmic concentrations covering three orders of magnitude, with three EXTs representing 3,333 molecules, three EXTs 1,054 molecules, three EXTs representing 333 molecules, and three EXTs representing 105 molecules. EXT sequences were PCR-amplified from cDNA samples using ‘decoding’ primer Dec1 and Dec2. For next-generation-sequencing Ion Torrent specific sequencing adapters were attached 5′ and 3′ to the amplified EXT sequences within a second round of PCR. On order to differentiate different samples 13mer barcodes were introduced between 5′ Ion adapter and target sequence. All processed samples of an experiment were combined within one pool and purified using PCR clean-up kit (Macherey-Nagel). The final samples were quantified using the dsDNA HS Assay Kit with the Qubit Fluorometer (Thermo Fisher Scientific) and adjusted to 23 pM. EXT samples were prepared and sequenced with the Ion Torrent Personal Genome Machine (PGM) system according to the manufacturer protocols for 200 bp template preparation and sequencing (Thermo Fisher Scientific).

### Data analysis of EXT reporter assays

Raw sequencing data analyses were done by using BLAST algorithm and in-house developed R scripts. First, on the basis of the sample barcodes the data were split into the original samples encoding the specific treatment conditions and the reads of the assay EXTs, eCals and iCals were counted to obtain total read numbers of the individual samples. Samples with total reads below 20% of the average were excluded from the experiment to avoid variability caused by low read samples. Total read distributions between sample codes were balanced by normalization of the individual read numbers per EXT and sample to the highest total read number to enable the comparison of EXT reads between samples (Supplementary Fig. S4a). The eCals are also used to compensate an overamplification of highly expressed EXTs within the library amplification PCRs and the suppression of effects of lower expressed EXTs. These effects can be detected in samples with low read numbers of the eCals. To balance these effects, all reads of a sample were calibrated to a normalization factor calculated from the eCal reads of the corresponding sample (Supplementary Fig. S4b,c). Following to normalization and calibration steps, the biological sample replicates and the three EXTs assigned to the GPCRs were consolidated, and the resulting samples were standardized to a reference sample. In GPCR activation EXT assays, all samples that contain a stimulus were standardized to the unstimulated control sample to calculate fold changes (FC) of receptor activation.

$$FC=\frac{stimulated\,condition\,mean}{unstimulated\,mean}$$

## Electronic supplementary material

Supplementary Information
