# Supplementary material for: Multiplexed profiling of GPCR activities by combining split TEV assays and EXT-based barcoded readouts
Source: Sci Rep. 2018 May 25;8:8137. doi: 10.1038/s41598-018-26401-9 (PMC5970223; doi:10.1038/s41598-018-26401-9)
Supplement: Supplementary file 1 — Supplementary Information [file 41598_2018_26401_MOESM1_ESM.pdf]

Supplementary Information

Multiplexed profiling of GPCR activities by combining split TEV assays and EXT-based barcoded readouts

Sabrina Galinski[1], Sven P. Wichert[1,2], Moritz J. Rossner[1] *, Michael C. Wehr[1] *

[1] Molecular Neurobiology, Department of Psychiatry, Ludwig Maximilian University of Munich, Germany, Nussbaumstr. 7, 80336 Munich

[2] Systasy Bioscience GmbH, Adams-Lehmann-Str. 56, 80797 Munich

* Corresponding authors:

Moritz.Rossner@med.uni-muenchen.de; phone: +49 (0)89 4400 55891

Michael.Wehr@med.uni-muenchen.de; phone: +49 (0)89 4400 53275

Supplementary Figures 3
Supplementary Tables 10
Supplementary Methods 16
Supplementary References 17

Supplementary Figures

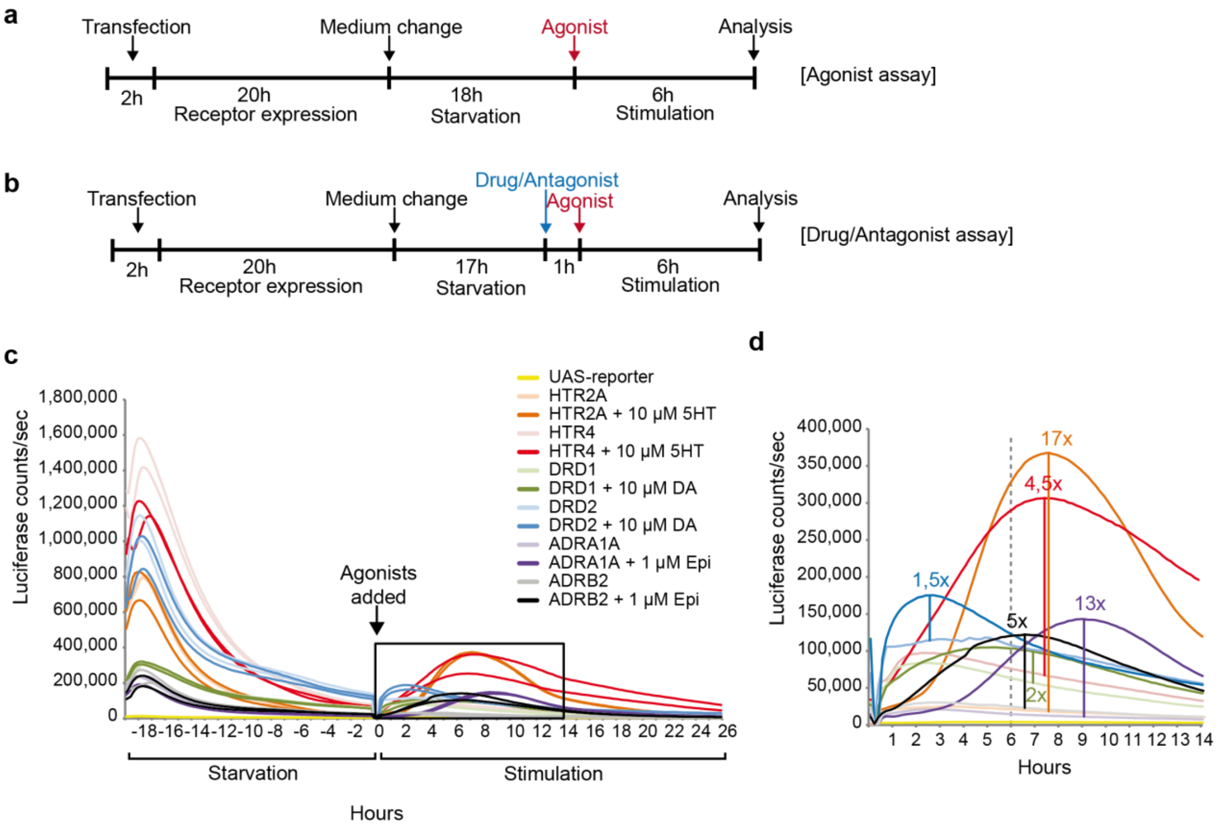

Supplementary Figure S1. Workflow and online monitoring of GPCR split TEV assays.

In all assay types cells were transiently transfected for 2h followed by an expression phase of 20h. Afterwards, the medium was changed by serum reduced assay medium for reducing basal cell activity (starvation period). (**a**) In agonist assays, an agonist was added and incubated for 6h before lysis. (**b**) In drug/antagonist assays, cells were pre-incubated with a drug/antagonist for 1h before adding the corresponding agonist for additional 6h followed by lysis. (**c**) For kinetic assays, agonists were added to cells, and cells were incubated in a lumicycler device (lumiCycle 32 from ActiMetrics) to monitor the luciferase activity over time. Receptor activation kinetics was measured for HTR2A, HTR4, DRD1, DRD2, ADRA1A and ADRB2. PC12 cells were transfected with 'GPCR'-V2R-NTEV-tevS-GV and ß-arrestin2delta-CTEV plasmids and the luciferase activity measured constantly during starvation phase (18h) and after application of agonists at indicated concentrations (stimulation phase, 26h). Decreasing signals during starvation phase reflect the reduction of cell activity. Luciferase readings of stimulation phase are highlighted in the box shown in (**d**). (**d**) Stimulation phase reads for each GPCR. Means of two replicates of unstimulated and stimulated samples are displayed; dotted line indicates reads at 6h post stimulation. Fold changes show peak levels for each GPCR. Agonists: 5HT, serotonin; DA, dopamine; Epi, epinephrine.

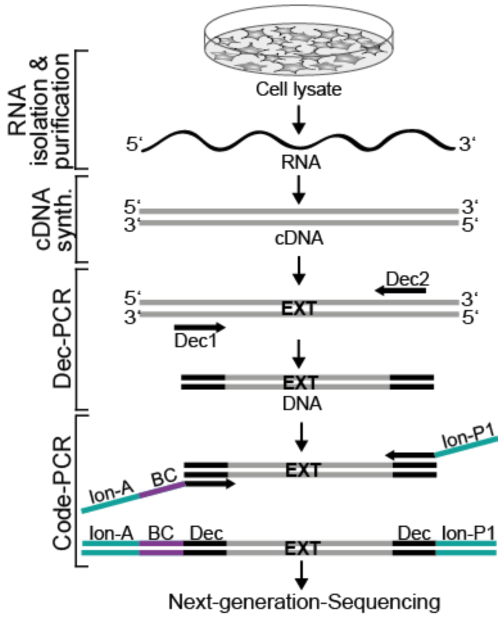

Supplementary Figure S2. Workflow of EXT sequencing library preparation.

Total RNA was isolated and purified from cell lysates and converted into cDNA by reverse transcription. EXT sequences were amplified by a decoding PCR (Dec-PCR) using Dec1 and Dec2 primer. Ion-A adapters together with unique 13mer barcodes (BC) and Ion-P1 adapters were added by Code-PCR.

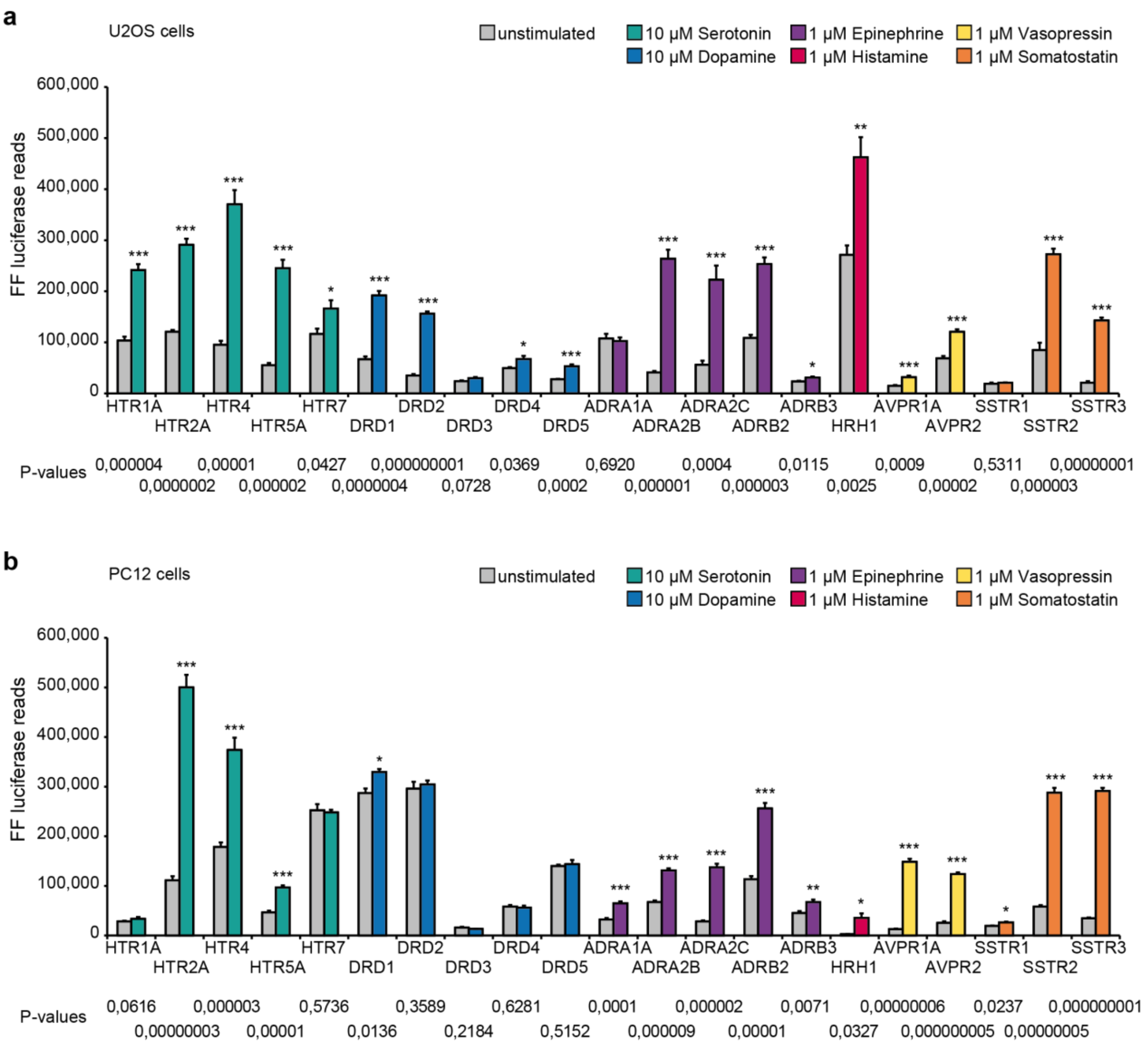

Supplementary Figure S3. Validation of single split TEV-based GPCR split TEV assays.

Single GPCR split TEV assays were performed as luciferase assays both in (**a**) U2OS and (**b**) PC12 cells. DRD3 did not show any significant activation and was not included into any further multiplexed GPCR assay. Grey bars indicate baseline activity levels, coloured bars indicate ligand-induced activity levels after 6h of stimulation. Colours define the subclasses of the GPCRs, with green for serotonin receptors, blue for dopamine receptors, violet for adrenergic receptors, red for histamine receptors, yellow for vasopressin receptors, and orange for somatostatin receptors. Data are shown as means ± s.e.m. (error bars), n=6. *p ≤ 0.05; **p ≤ 0.01; *p ≤ 0.001 (two-sided t-test).

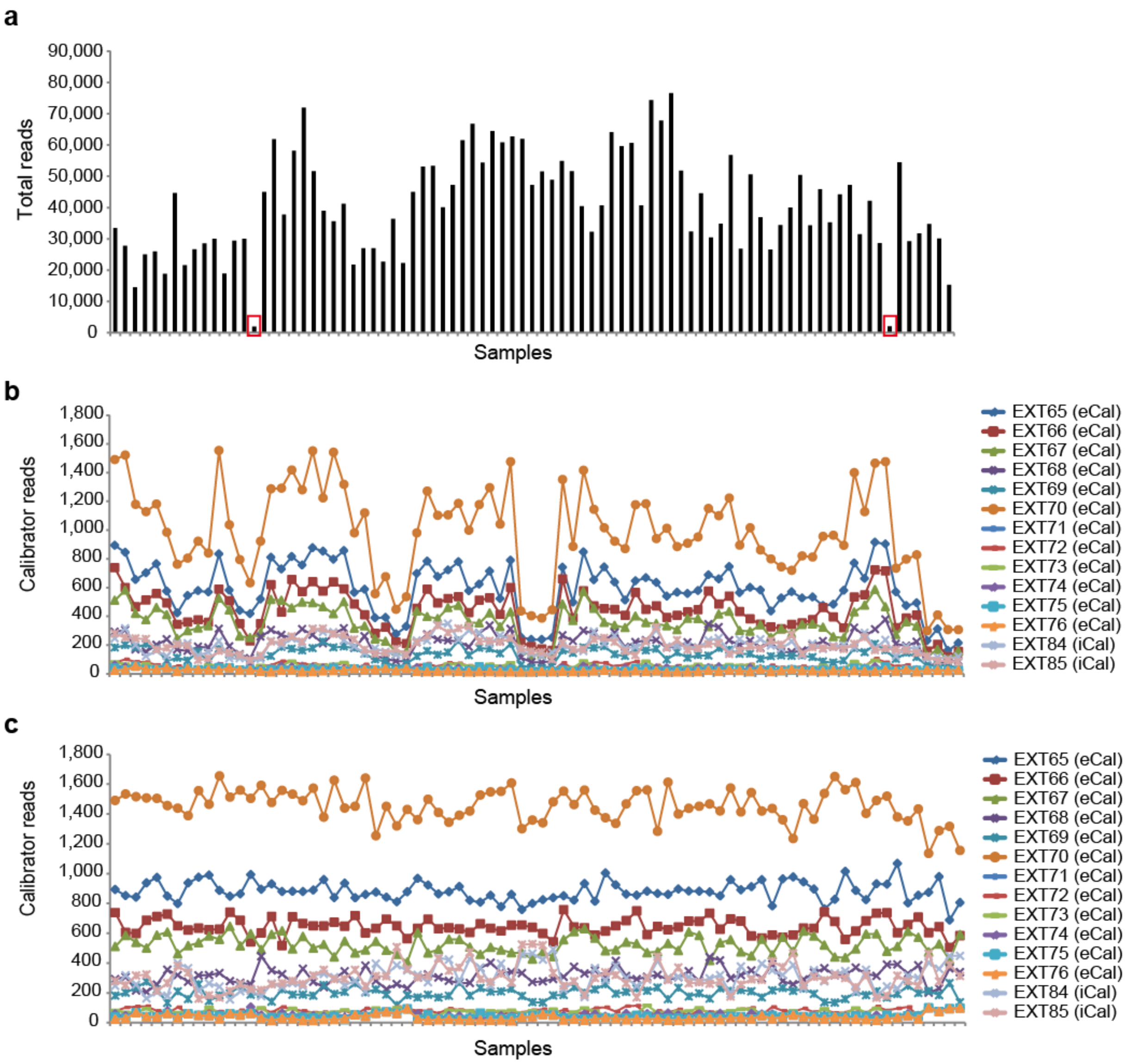

Supplementary Figure S4. Processing of sequencing data.

(**a**) Total reads per sample of raw sequencing data. Samples with a total read value below the threshold were excluded from further analysis (samples are marked by red boxes). Bottom, total reads per sample after normalization. (**b**) Reads of external (eCal) and internal (iCal) calibrator EXTs after normalization to total reads. Normalization factor was calculated from the highest mean of samples. (**c**) Calibrator EXT reads after application of normalization factor.

a

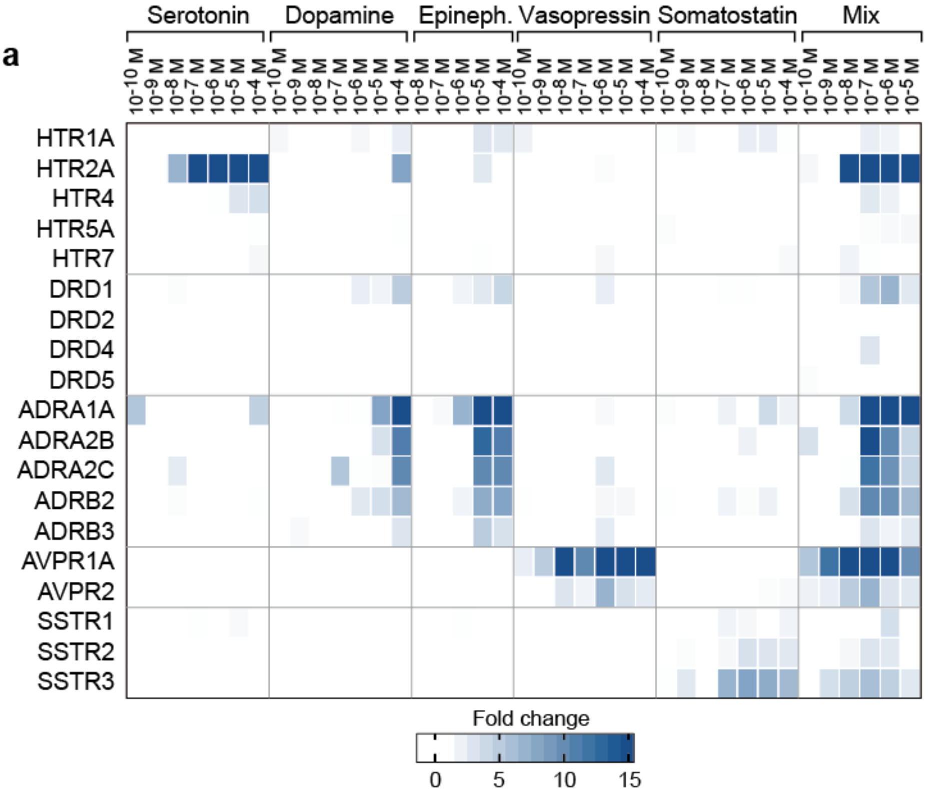

b

	5-HT		DA		Epi		AVP		SST		Mix	
	U2OS	PC12	U2OS	PC12	U2OS	PC12	U2OS	PC12	U2OS	PC12	U2OS	PC12
HTR1A				2,2		3,0		2,0		2,2		2,2
HTR2A	3,9	89,5		8,2		2,7					4,2	273,4
HTR4	4,9	3,6									3,6	2,7
HTR5A	3,1		2,7								4,2	
HTR7	2,0											
DRD1				5,1	2,0	4,3		2,2				7,0
DRD2	3,6		5,1		5,0						3,6	
DRD4			5,0		3,2						2,8	3,0
DRD5			2,2		2,5							
ADRA1A		5,5		83,4	2,5	314,2				3,9		361,0
ADRA2B			5,6	11,1	9,2	12,9				2,0	6,3	16,1
ADRA2C		2,5	11,2	10,3	13,5	10,3		2,7			11,3	12,0
ADRB2				6,5	2,7	8,2				2,0	2,2	10,3
ADRB3				3,0		5,1		2,5				3,0
AVPR1A							3,1	44,3			2,8	47,5
AVPR2								7,0				7,0
SSTR1									2,7		2,8	3,6
SSTR2							2,8		7,5	3,3	6,9	3,0
SSTR3									9,1	8,2	9,3	6,0

Fold change

< 2	> 2	> 4	> 6	> 10	> 15	> 100

Supplementary Figure S5. EXT-based GPCR profiles show cell-type dependent activities.

(**a**) Heat map of multiplexed GPCR activation in PC12 cells. Plotted are fold changes of receptor activation in reference to unstimulated condition of 19 different GPCRs. GPCRs were stimulated with the single compounds serotonin, dopamine, epinephrine, vasopressin and somatostatin and a mixture (Mix) of the five compounds. To visualize lower effects the colour scale is limited to 15-fold changes. (**b**) Summary of GPCR activations measured in U2OS and PC12 cells. Shown are the highest fold changes of each GPCR agonist interaction obtained in multiplexed profiling assays.

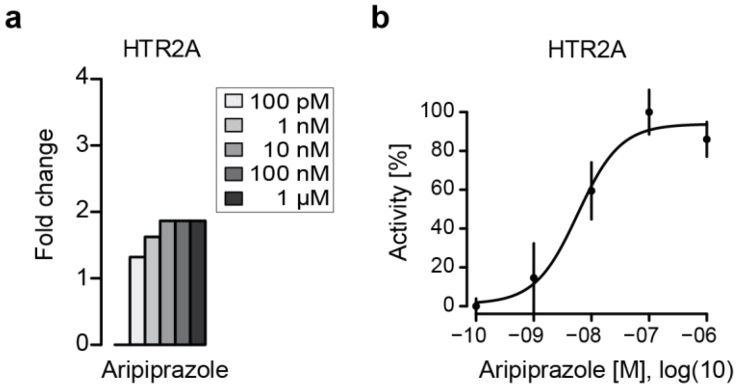

Supplementary Figure S6. Dose-dependent activation of HTR2A by aripiprazole.

Aripiprazole shows concentration-dependent agonistic effect on the serotonin receptor HTR2A in the multiplexed GPCRprofiler assay. (**a**) Plotted are fold changes of receptor activation in reference to unstimulated condition of the serotonin receptor HTR2A by aripiprazole. (**b**) Corresponding dose-response curve of receptor activation, data are shown as mean ± s.e.m. (error bars), n=6.

Supplementary Tables

Supplementary Table S1.

Table listed all GPCRs and the assigned EXTs used in GPCRprofiler assays as denoted in the main text, the full fusion protein constructs, and the corresponding Reference Sequence (RefSeq) numbers.

Receptor subgroup	GPCR (denoted in text)	Full fusion protein	RefSeq	EXTs assigned to experiments		
				Agonist assay U2OS cells	Agonist assay PC12 cells	Drug assay U2OS cells
Serotonin receptors	HTR1A	HA-FLAG-HR1A-V2R-NTEV-tevS-GV	NM_000524	EXT01	EXT01	EXT01
				EXT02	EXT02	EXT02
				EXT03	EXT03	EXT03
	HTR2A	HOOK-HTR2A-V2R-NTEV-tevS-GV	NM_000621	EXT04	EXT04	EXT04
				EXT05	EXT05	EXT05
				EXT06	EXT06	EXT06
	HTR4	HTR4-V2R-NTEV-tevS-GV	NM_001040169	EXT07	EXT07	EXT07
				EXT08	EXT08	EXT08
				EXT09	EXT09	EXT10
	HTR5A	HTR5A-V2R-NTEV-tevS-GV	NM_024012	EXT10	EXT10	EXT11
				EXT11	EXT11	EXT12
				EXT12	EXT12	EXT13
	HTR7	HA-FLAG-HTR7-V2R-NTEV-tevS-GV	NM_000872	EXT13	EXT13	*
				EXT14	EXT14	
				EXT15	EXT15	
Dopamine receptors	DRD1	DRD1-V2R-NTEV-tevS-GV	NM_000794	EXT16	EXT16	EXT18
				EXT17	EXT17	EXT19
				EXT18	EXT18	EXT20
	DRD2	DRD2-V2R-NTEV-tevS-GV	NM_016574 (short isoform)	EXT19	EXT19	EXT21
				EXT20	EXT20	EXT80
				EXT21	EXT21	EXT81
	DRD4	HA-FLAG-DRD4-V2R-NTEV-tevS-GV	NM_000797	EXT22	EXT22	EXT24
				EXT23	EXT23	EXT25
				EXT24	EXT24	EXT26
	DRD5	DRD5-V2R-NTEV-tevS-GV	NM_000798	EXT25	EXT25	EXT27
				EXT26	EXT26	EXT28
				EXT27	EXT27	EXT29
α-Adrenergic receptors	ADRA1A	ADRA1A-V2R-NTEV-tevS-GV	NM_033303	EXT28	EXT28	EXT30
				EXT29	EXT29	EXT31
				EXT30	EXT30	EXT32
	ADRA2B	ADRA2B-V2R-NTEV-tevS-GV	NM_000682	EXT31	EXT31	EXT33
				EXT32	EXT32	EXT34
				EXT33	EXT33	EXT35
	ADRA2C	ADRA2C-V2R-NTEV-tevS-GV	NM_000683	EXT34	EXT34	EXT36
				EXT35	EXT35	EXT37
				EXT36	EXT36	EXT38
β-Adrenergic receptors	ADRB2	ADRB2-V2R-NTEV-tevS-GV	NM_000024	EXT37	EXT37	EXT39
				EXT38	EXT38	EXT40
				EXT39	EXT39	EXT41
	ADRB3	ADRB3-V2R-NTEV-tevS-GV	NM_000025	EXT40	EXT40	*
				EXT41	EXT41	
				EXT42	EXT42	

Histamine receptor	HRH1	HRH1-V2R-NTEV-tevS-GV	NM_000861	*	*	EXT42
						EXT43
						EXT44
Vasopressin receptors	AVPR1A	AVPR1A-V2R-NTEV-tevS-GV	NM_000706	EXT43	EXT43	EXT45
				EXT44	EXT44	EXT46
				EXT45	EXT45	EXT47
	AVPR2	AVPR2-V2R-NTEV-tevS-GV	NM_000054	EXT46	EXT46	EXT48
				EXT47	EXT47	EXT49
				EXT48	EXT48	EXT50
Somatostatin receptors	SSTR1	SSTR1-V2R-NTEV-tevS-GV	NM_001049	EXT49	EXT49	EXT51
				EXT50	EXT50	EXT52
				EXT51	EXT51	EXT53
	SSTR2	SSTR2-V2R-NTEV-tevS-GV	NM_001050	EXT52	EXT52	EXT54
				EXT53	EXT53	EXT55
				EXT54	EXT54	EXT56
	SSTR3	SSTR3-V2R-NTEV-tevS-GV	NM_001051	EXT55	EXT55	EXT57
				EXT56	EXT56	EXT58
				EXT57	EXT57	EXT59

*: GPCR not included in experiment

Supplementary Table S2.

Sequences of all EXT barcodes used in the assays.

EXTs	Sequence
EXT01	AATCCAAATCTACTTTACATTGTGGCTCTACATCTTTATCATACTTCTA
EXT02	ACATCTTTAATCACATCAAATGTGGACACAAATTACATCAACATACAT
EXT03	CAAATACTTTACCTTTCTTTACAGCGACTCTTTCAAATCTATACTCTTT
EXT04	ATCACTTTAATCCTTTTCTATCACGGTGTTCTAACATATCATTACTCTA
EXT05	TTACTACTCTTTTACTACATTCTCGGTGTACATATCAATCAAATCACAT
EXT06	TACTCAAATACTTCTACTTTACAGGCTGACTTTTACTTCTATACTACAT
EXT07	ATCATCTACAAAACATTCTAACAGCCTGATCTATCTACAAACTTTCAAA
EXT08	AATCACATTTACCTTTTCTATGACGGAGTTCTAACATATCATCTACTTT
EXT09	TTACCTTTACATACATACATAGACCCTCAACATAATCCTTTTCTACAAA
EXT10	TACTATCATACTCTTTCTTTAGAGCCTGACTTTTCTATTACACATATCA
EXT11	ATCAACATTACTACATCAAAAGTGGGTGTCAAAACATCTTTACATACAT
EXT12	ATCACTTTAATCTTACTCTAAGAGCGTCATCTATACTATCATTACTACT
EXT13	TACTATCACAAATTACCAAATGTCGCTCTCAAACTTTTACTTCTATACT
EXT14	ACATTCTACTTTTACTCTTTACAGCCACACTTTTCTAAATCAATCTCTA
EXT15	ACATTCTATTACCAAATCTATGAGCGTCATCTAACATCAAAAATCCTTT
EXT16	TCTATACTCAAAACATACATTCAGCCACAACATTACTATCAAATCCAAA
EXT17	ATCAACATTACTATCATCTATGTCCGTCATCTATTACTCTATCTATCTA
EXT18	ATCAATCATTACTCTACAAATCTGGCAGACAAATTACTCTACAAATCTA
EXT19	CTTTACATTCTATCTACTTTACTGCGTGACTTTTTACCAAAAATCATCA
EXT20	ACATCTTTCTTTCAAACTTTACACCGTCTCTTTAATCCTTTACATACAT
EXT21	CTTTACATTACTACATACATACACCGTGTACATATCAACATAATCACAT
EXT22	TTACATCATCTATACTACATTGTCCCTCAACATATCAATCATCTATCTA
EXT23	TACTACATATCAATCATCTATCTCCGACTTCTACAAATCTACAAACTTT
EXT24	TACTTCTATACTTTACTCTATGAGCGTGTTCTATACTCTTTCTTTATCA
EXT25	TTACAATCTCTCTCTATCTATCACGGACATCTAAATCATCATCTACTTT
EXT26	CTTTTCTAATCAATCACTTTAGTGGGTGTCTTTTCTATACTTTACTACT
EXT27	ATCATTACAATCACATACATTCAGCCTGAACATTACTATCATCTACTTT
EXT28	ACATTCTACTTTACATACATAGAGGGTGTACATATCAAATCCAAAATCA
EXT29	AATCTCTATTACACATCAAAAGACGCTGACAAATCTAATCAATCAATCA
EXT30	ATCATACTTTACATCACTTTTCTGCCTGACTTTTTACCTTTACATATCA
EXT31	TACTACATCTTTTCTATCTAAGAGGGTGTTCTAATCAATCACTTTATCA
EXT32	TTACCAAATACTTTACCTTTTGAGCGACTCTTTCTTTTTACATCATTAC
EXT33	TCTATCTATCTATACTCAAATCTCGGTCTCAAATTACAATCTACTTCTA
EXT34	TCTATACTTACTTCTAACATTGACCGTCTACATTACTTCTATCTATTAC
EXT35	AATCCAAATACTTTACTCTATGTCCGACTTCTATACTTACTCTTTATCA
EXT36	TCTATACTACATCTTTTCTAAGACGGTGTTCTAAATCAATCACATCTTT
EXT37	ATCAACATCTTTCTTTACATACTGGGAGTACATCTTTACATATCATTAC
EXT38	TCTACAAATACTTTACTCTATGTCCCTCTTCTACAAATACTTACTTCTA
EXT39	ATCAATCACAAATCTATCTAACACCGTGTTCTATTACAATCCTTTTTAC
EXT40	CTTTTACTCAAAAATCACATACAGGGTCAACATCTTTCTTTATCACAAA
EXT41	ATCAACATAATCACATCTTTAGAGCCTGACTTTACATTACTTACTACAT
EXT42	ATCAATCATTACACATCAAATGTCGGTCTCAAAACATTACTACATAATC
EXT43	AATCATCATTACACATTCTATCAGGCTGTTCTAATCAATCATCTAATCA
EXT44	ATCATACTCTTTTACTCAAAAGTGGCTCTCAAAATCAAATCAATCACAT
EXT45	TCTATACTATCAACATCTTTAGACCGTGTCTTTTCTATACTAATCTTAC
EXT46	AATCATCATTACTCTACTTTAGACCGAGTCTTTCTTTACATTACTTTAC
EXT47	CTTTTACTATCATACTCTTTTCAGCTTCACTTTCTTTTTACTCTACAAA
EXT48	TACTTACTACATCAAAACATAGACCGAGTACATTCTAAATCCTTTTACT
EXT49	ATCATCTAATCAACATACATAGTGGGAGAACATTCTAATCAAATCTCTA
EXT50	ACATTTACACATCAAACAAATGACCGTCACAAAATCAACATCTTTACAT
EXT51	TCTAATCAAATCACATTCTAACTGCGACATCTAACATATCATCTATTAC

EXT52	CTTTTCTAACATTCTATCTATGTCCCAGATCTATCTACTTTTACTTTAC
EXT53	TTACTACTACATACATACATTCAGCCTCAACATCAAAAATCCTTTCTTT
EXT54	TTACATCAAATCCTTTACATACTGGGACAACATCTTTAATCCAAAATCA
EXT55	CTTTTCTACTTTTTACACATTGTGGGAGAACATTACTATCAAATCATCA
EXT56	ACATACATACATTTACACATTCAGGGACAACATTCTACAAATTACCAAA
EXT57	TACTTTACCAAACTTTTCTAAGACGCTCATCTAATCACAAAAATCACAT
EXT58	TACTTCTACTTTACATCTTTAGTGCGTCTCTTTTCTATTACAATCTACT
EXT59	TTACCAAAACATTTACACATTGACGGTGTACATATCATCTATACTAATC
EXT60	ATCAAATCCTTTAATCACATTCTGGCTCAACATATCATTACACATCAAA
EXT61	AATCATCAATCATACTCTTTTCTGGCTCTCTTTTTACCAAACTTTCTTT
EXT62	CAAAACATTACTACATTCTAAGTGGCTCTTCTAATCATACTTCTACAAA
EXT63	AATCACATTACTAATCACATACACGGTGAACATACATCAAAATCACAAA
EXT64	CTTTTACTTCTAACATCTTTAGTGCCTGACTTTTCTATACTAATCAATC
EXT65	ACATACATCAAAACATCTTTAGAGCCTGTCTTTCTTTATCAACATTACT
EXT66	ACATATCAACATTCTACTTTTGTGCGTGACTTTTTACTACTTACTACAT
EXT67	ACATAATCCTTTCTTTACATACACGGAGTACATTACTACATTACTTTAC
EXT68	TACTTCTACATTGTCCCTCAACATTCTACTTTTCTACTTTCCTATAGTG
EXT69	ATCATCTAACATCAAACTTTTGAGGCTGACTTTCTTTTTACACATTTAC
EXT70	ATCATCTACAAATCTAACATTCTGGCTCTACATATCAACATTACTTCTA
EXT71	CAAACTTTTTACATCACAAAACTCGCAGACAAAACATAATCTTACCAAA
EXT72	CAAATCTATACTCAAAACATAGTGGGTGAACATTACTTACTATCATCTA
EXT73	CAAAATCATTACATCAACATACACCGTGTACATTCTAACATATCACTTT
EXT74	ACATTCTAAATCACATCAAAACTCGGTGACAAACTTTACATTTACACAT
EXT75	ACATATCAACATAATCTCTATCAGCCACATCTAATCATACTAATCTCTA
EXT76	CAAATTACCAAACTTTTCTATCTGGGTGTTCTACAAACTTTCTTTTTAC
EXT77	ATCATTTAACATAATCTCTATGACGGTCTTCTAATCATTACATCACAAA
EXT78	AATCTACTTACTTCTACAAATCACGGACACAAATTACTCTAATCACTTT
EXT79	TTACCTTTACATACATACATTCAGCGTGTACATTCTACTTTCTTTAATC
EXT80	AATCTCTACAAACAAATCTATCAGGCTGATCTAACATCTTTTTACCTTT
EXT81	ATCAATCAAATCTCTATCTAAGACGGAGATCTAAATCATCATACTTCTA
EXT82	ACATCAAATACTTCTATCTATGAGGCTGATCTAAATCCTTTACATTACT
EXT83	TCTAACATATCATCTAACATAGAGGGTCAACATACATTCTAAATCAATC
EXT84	AATCCAAACTTTATCAACATTCAGCGACTACATACATCAAAAATCAATC
EXT85	TCTATACTTTACTACTTCTAACAGGCACATCTAATCAAATCAATCACAT

Supplementary Table S3.

Overview of the experimental conditions of the different types of assays.

	**Luciferase assay**	**Single EXT assay**	**Multiplexed EXT assay (GPCRprofiler)**	**Kinetic assay**
**Plate format**	96-well	6-well	6-well	3.5 cm dish
**Cell number per well**	4×10^4 (PC12)	4×10^5 (PC12)	6×10^5 (U2OS)<br>1.4×10^6 (PC12)	1×10^6 (PC12)
**Transfection**	on-plate	on-plate	in-solution	on-plate
**Lysis buffer**	PLB	RLT	RLT	-
**Lysis volume**	30 µl	500 µl	500 µl	-

Supplementary Table S4.

Comparison of drug-induced effects measured by the GPCRprofiler and the public databases PubChem[1] and IUPHAR[2,3].

	Paliperidone			Aripiprazole		
**Receptor**	**GPCRprofiler**	**PubChem**	**IUPHAR**	**GPCRprofiler**	**PubChem**	**IUPHAR**
**HTR2A**	Antagonist	Antagonist[4–6]	Antagonist[7]	Agonist	Antagonist[8,9]	Agonist[10,11]
**DRD2**	Antagonist	Antagonist[4,5]	N.A.	Agonist	Antagonist/ partial agonist[12–14]	Agonist[15,16]
**ADRA2B**	Antagonist	Antagonist[6]	N.A.	N.A.	Antagonist[17]	N.A.
**ADRA2C**	Antagonist	N.A.	N.A.	Antagonist	Antagonist[17]	N.A.
**HRH1**	Antagonist	Agonist[4–6]	Antagonist[7]	Antagonist	Antagonist[17]	Antagonist[10]

N.A., not available.

Supplementary Methods

Online luciferase reporter assays

For online GPCR split TEV assays PC12 cells were plated on 3.5 cm dishes at 1 x 10^6 cells per dish. On the next day, cells were transfected with lipofectamine 2000 (Invitrogen) diluted in opti-MEM (Gibco) and incubated for 2h at 37°C, followed by exchange of transfection medium by culture medium (low glucose DMEM medium (1 g/L, Lonza), supplemented with 10% FBS, 5% HS, 2 mM GlutaMAX (Gibco) and 100 U/ml each of penicillin and streptomycin). After 24h medium was exchanged by starvation medium (low glucose DMEM (1 g/L) supplemented with 1% FBS and 0.1 mM NEAA) additionally supplemented with 0.1% luciferin (Promega). To measure the firefly luciferase activity, the dishes were transferred to a 32-channel luminometer (lumiCycle 32 by ActiMetrics), which was located inside a cell culture incubator at 37°C and 5% CO_2. Cells were starved for 16h followed by compound treatment and online luciferase readings over 36h.

Modification of GPCR signal peptides

Due to a low expression rate and functionality in split TEV assays of the serotonin receptors HTR1A, HTR2A and HTR7, and the dopamine receptor DRD4 the signal peptide for cell membrane localization was either exchanged or an additional signal peptide was added. For the serotonin receptors HTR1A and HTR7 and the dopamine receptor DRD4 a HA-Flag signal peptide (sequence: ATGGAGACAGACACACTCCTGCTATGGGTACTGCTGCTCTGGGTTCCAGGTTCCACTGGTGAC) was added by PCR N-terminal in front of the GPCR. For the modification of the serotonin receptor HTR2A by changing the signal peptide the native signal peptide sequence was determined using the program http://www.predisi.de/. The signal peptide was changed by the signal sequence of the mouse Ig κ-chain V-J2-C proteins (sequence: ATGGAGACAGACACACTCCTGCTATGGGTACTGCTGCTCTGGGTTCC AGGTTCCACTGGTGAC).

Supplementary References

1. Kim, S. *et al.* PubChem Substance and Compound databases. *Nucleic Acids Res.* **44,** D1202–D1213 (2016).
2. Harding, S. D. *et al.* The IUPHAR/BPS Guide to PHARMACOLOGY in 2018: updates and expansion to encompass the new guide to IMMUNOPHARMACOLOGY. *Nucleic Acids Res.* (2017). doi:10.1093/nar/gkx1121
3. Alexander, S. P. *et al.* THE CONCISE GUIDE TO PHARMACOLOGY 2017/18: G protein-coupled receptors. *Br. J. Pharmacol.* **174,** S17–S129 (2017).
4. Cohen, L. J. Risperidone. *Pharmacotherapy* **14,** 253–265 (1994).
5. Megens, A. A. *et al.* Survey on the pharmacodynamics of the new antipsychotic risperidone. *Psychopharmacology (Berl.)* **114,** 9–23 (1994).
6. Richelson, E. & Souder, T. Binding of antipsychotic drugs to human brain receptors focus on newer generation compounds. *Life Sci.* **68,** 29–39 (2000).
7. Schotte, A. *et al.* Risperidone compared with new and reference antipsychotic drugs: in vitro and in vivo receptor binding. *Psychopharmacology (Berl.)* **124,** 57–73 (1996).
8. Bortolozzi, A., Díaz-Mataix, L., Toth, M., Celada, P. & Artigas, F. In vivo actions of aripiprazole on serotonergic and dopaminergic systems in rodent brain. *Psychopharmacology (Berl.)* **191,** 745–758 (2007).
9. Stark, A. D. *et al.* Interaction of the novel antipsychotic aripiprazole with 5-HT1A and 5-HT 2A receptors: functional receptor-binding and in vivo electrophysiological studies. *Psychopharmacology (Berl.)* **190,** 373–382 (2007).
10. Kroeze, W. K. *et al.* H1-histamine receptor affinity predicts short-term weight gain for typical and atypical antipsychotic drugs. *Neuropsychopharmacol. Off. Publ. Am. Coll. Neuropsychopharmacol.* **28,** 519–526 (2003).

11. Shapiro, D. A. *et al.* Aripiprazole, a novel atypical antipsychotic drug with a unique and robust pharmacology. *Neuropsychopharmacol. Off. Publ. Am. Coll. Neuropsychopharmacol.* **28,** 1400–1411 (2003).

12. Hirose, T. & Kikuchi, T. Aripiprazole, a novel antipsychotic agent: dopamine D2 receptor partial agonist. *J. Med. Investig. JMI* **52 Suppl,** 284–290 (2005).

13. Wood, M. & Reavill, C. Aripiprazole acts as a selective dopamine D2 receptor partial agonist. *Expert Opin. Investig. Drugs* **16,** 771–775 (2007).

14. Wood, M. D. *et al.* Aripiprazole and its human metabolite are partial agonists at the human dopamine D2 receptor, but the rodent metabolite displays antagonist properties. *Eur. J. Pharmacol.* **546,** 88–94 (2006).

15. Allen, J. A. *et al.* Discovery of β-Arrestin-Biased Dopamine D2 Ligands for Probing Signal Transduction Pathways Essential for Antipsychotic Efficacy. *Proc. Natl. Acad. Sci. U. S. A.* **108,** 18488–18493 (2011).

16. Zajdel, P. *et al.* Antidepressant and antipsychotic activity of new quinoline- and isoquinoline-sulfonamide analogs of aripiprazole targeting serotonin 5-HT_1A/5-HT_2A/5-HT_7 and dopamine D_2/D_3 receptors. *Eur. J. Med. Chem.* **60,** 42–50 (2013).

17. Nasrallah, H. A. Atypical antipsychotic-induced metabolic side effects: insights from receptor-binding profiles. *Mol. Psychiatry* **13,** 27–35 (2008).
